# Effect of droplet size on the encapsulation efficiency of microparticles in passive microfluidic systems

**DOI:** 10.1007/s10544-026-00795-0

**Published:** 2026-03-31

**Authors:** Risa Fujita, Masashi Kobayashi, Takashi Tanii, Masahiro Furuya, Shuichi Shoji, Daiki Tanaka

**Affiliations:** 1 Research Organization for Nano & Life Innovation, Waseda University, Tokyo, 162-0041 Japan; 2 Faculty of Science and Engineering, Waseda University, Tokyo, 169-8555 Japan

**Keywords:** Encapsulation, Microfluidics, Droplet, Poisson distribution

## Abstract

Droplet microfluidic technologies are widely used in single-cell analysis, in which passive encapsulation methods are commonly used to isolate individual cells or particles. In these systems, the number of particles per droplet is assumed to follow a Poisson distribution. However, the extent to which droplet size affects encapsulation behavior under passive conditions is poorly understood. In this study, we systematically investigated the effect of droplet diameter on single-particle encapsulation fidelity by generating agarose droplets 30, 50, and 100 μm using a flow-focusing microfluidic device. Fluorescent beads were encapsulated at a concentration corresponding to a mean occupancy of 1, and encapsulation statistics were analyzed across droplet sizes. The 30 and 50 μm droplets exhibited encapsulation distributions closely aligned with the Poisson model, whereas 100 μm droplets showed a large excess of empty droplets and substantial deviation from the expected profile. To examine whether comparable size-dependent trends are observed in a biological sample, green fluorescent protein-expressing *Escherichia coli* were encapsulated in droplets of the same sizes. The 30 μm droplets consistently showed the closest agreement with Poisson statistics, whereas the 50 and 100 μm droplets showed comparable encapsulation fidelity, with moderate deviations and a modest increase in empty droplets at the largest diameter. These findings indicate that smaller droplets provide robust stochastic encapsulation in passive systems, while larger droplets may exhibit reduced fidelity depending on particle type. Our results suggest that droplet size is an important factor to consider when designing simplified and reliable microfluidic systems for biological applications.

**Supplementary Information:**

The online version contains supplementary material available at 10.1007/s10544-026-00795-0.

## Introduction

Droplet microfluidics technology is used to manipulate discrete volumes of fluid in immiscible phases. Water-in-oil droplets are microscale aqueous compartments, which are formed by injecting two immiscible fluids, namely the continuous and dispersed phases, into a microfluidic channel. The shear force of the continuous phase, typically oil, generates stable droplets from the dispersed phase. The droplets can function as individual microreactors, and if the dispersed phase contains particles, such as cells, nucleic acids, microorganisms, or beads, encapsulation occurs, creating droplets that contain particles. Encapsulation enables microscale reactions and analysis of individual cell characteristics under highly controllable and reproducible conditions. Furthermore, processing a large number of droplets in parallel facilitates high-throughput analysis.

This unique capability of droplet microfluidics technology has led to a wide range of biological and biomedical applications, including on-chip analysis, cell culture, drug screening and diagnostics, and single-cell analysis (Mashaghi et al. 2016; Amirifar et al. 2022; Nan et al. 2024). Among these, droplet microfluidic PCR has been a landmark development (Beer et al. 2007). Because the reaction volume within each droplet is tiny, droplet microfluidic PCR requires minimal amounts of template DNA, allowing sensitive detection (Zhang and Jiang 2016). For instance, Bian et al. (2015) reported a droplet digital PCR platform based on an oil-saturated polydimethylsiloxane (PDMS) chip that detected pathogenic bacteria with a low detection limit. Droplet microfluidics technology is also used for small-scale cell culture, in which one to several hundred cells are encapsulated in water-in-oil emulsions, allowing different types of culture to be applied to small numbers of cells, spheroids, and organoids (Clausell-Tormos et al. 2008; Sart et al. 2022; Wang et al. 2023). Encapsulating cells in core-shell gel droplets makes them less susceptible to external environmental influences and promotes cell growth (Wang et al. 2019). In drug screening and disease diagnosis, microfluidic technology enables highly sensitive, high-throughput detection and screening of biomarkers by dividing bulk samples into many small droplets (Kaushik et al. 2018). Guan et al. (2015) developed an enzyme-linked oligonucleotide hybridization assay in which magnetic beads for capturing individual RNA molecules were encapsulated into droplets, allowing bacterial rRNA to be detected and quantified with high accuracy.

Over the past decade, single-cell analysis has advanced hugely with the development of methods for encapsulating individual targets within droplets (Wen et al. 2016; Jiang et al. 2023; Kumari et al. 2023). Single-cell analysis has contributed greatly to the study of biological processes and cellular heterogeneity, including gene expression analysis and proteomics. For instance, Klein et al. (2015) introduced the inDrop method, a droplet microfluidic platform, that enabled massively parallel single-cell RNA sequencing of thousands of cells with high throughput. Lan et al. (2017) conducted ultra-high throughput single-cell genome sequencing using droplet microfluidics. Cells were encapsulated in hydrogel microspheres, which enabled the isolation and barcoding of the genome within the droplets. This method facilitated the processing of over 50,000 bacterial and fungal cells in a few hours. In addition to analyzing single targets, droplets can also be used to compartmentalize pairs of individual cells and study their interactions (Tumarkin et al. 2011). For instance, single-cell pairs have been cocultured in droplets to identify and study cell–cell interactions in the central nervous system (Madrigal et al. 2022; Wheeler et al. 2023).

Droplets are generated by the passive method, which does not require an external energy source, or by the active method, which requires external actuation (Baroud et al. 2010; Zhu and Wang 2016; Chen et al. 2022). The criteria for cell encapsulation include high encapsulation efficiency, which implies a low rate of empty droplets and control of the number of cells per droplet. To achieve this, active approaches, such as acoustic manipulation and dielectrophoresis, have been employed either to deterministically align particles prior to droplet formation or to remove empty droplets after droplet generation (Isozaki et al. 2020; Link et al. 2021; He et al. 2025). Although these methods are effective, they often involve complex device architectures, external actuators, and precise flow control, which can pose challenges for routine biological assays.

In contrast, passive encapsulation strategies offer operational simplicity and device accessibility by relying solely on laminar flow and interfacial tension without external fields. In such systems, the encapsulation efficiency is governed primarily by particle concentration and droplet volume particle encapsulation inherently follows the Poisson distribution according to formula (1), (Sykes et al. 1992; Clausell-Tormos et al. 2008; Collins et al. 2015).

1 $$\:p\left(k\right)=\frac{{\lambda\:}^{k}{e}^{-{\uplambda\:}}}{k!}$$

Here, λ is the average number of particles per droplet and $\:k$ is the number of particles in a droplet. For instance, when the concentration of the solution is adjusted to ensure that one particle is contained in each droplet, the encapsulation efficiency expected from the Poisson distribution is approximately 36%. However, although the dependence of encapsulation efficiency on particle concentration is well studied, the effect of droplet diameter with the same mean occupancy remains insufficiently characterized.

In this study, we systematically investigated how droplet size affects Poisson-based encapsulation behavior using a passive flow-focusing microfluidic device. Agarose droplets with nominal diameters of 30, 50, and 100 μm were generated, and fluorescent microparticles were encapsulated at a concentration corresponding to a target Poisson parameter of λ = 1. The agreement between experimental encapsulation distributions and Poisson predictions was quantitatively evaluated using the coefficient of determination (*R*^2^) and the mean absolute error (MAE). In addition, encapsulation experiments using green fluorescent protein (GFP)-expressing *E. coli* were conducted under the same conditions to examine whether comparable size-dependent trends are observed in a biological sample. These results provide insights into size-dependent encapsulation behavior in passive systems and offer practical guidance for optimizing droplet-based assays such as digital PCR and single-cell analysis.

## Methods

### Device design and fabrication process

**Fig. 1:**
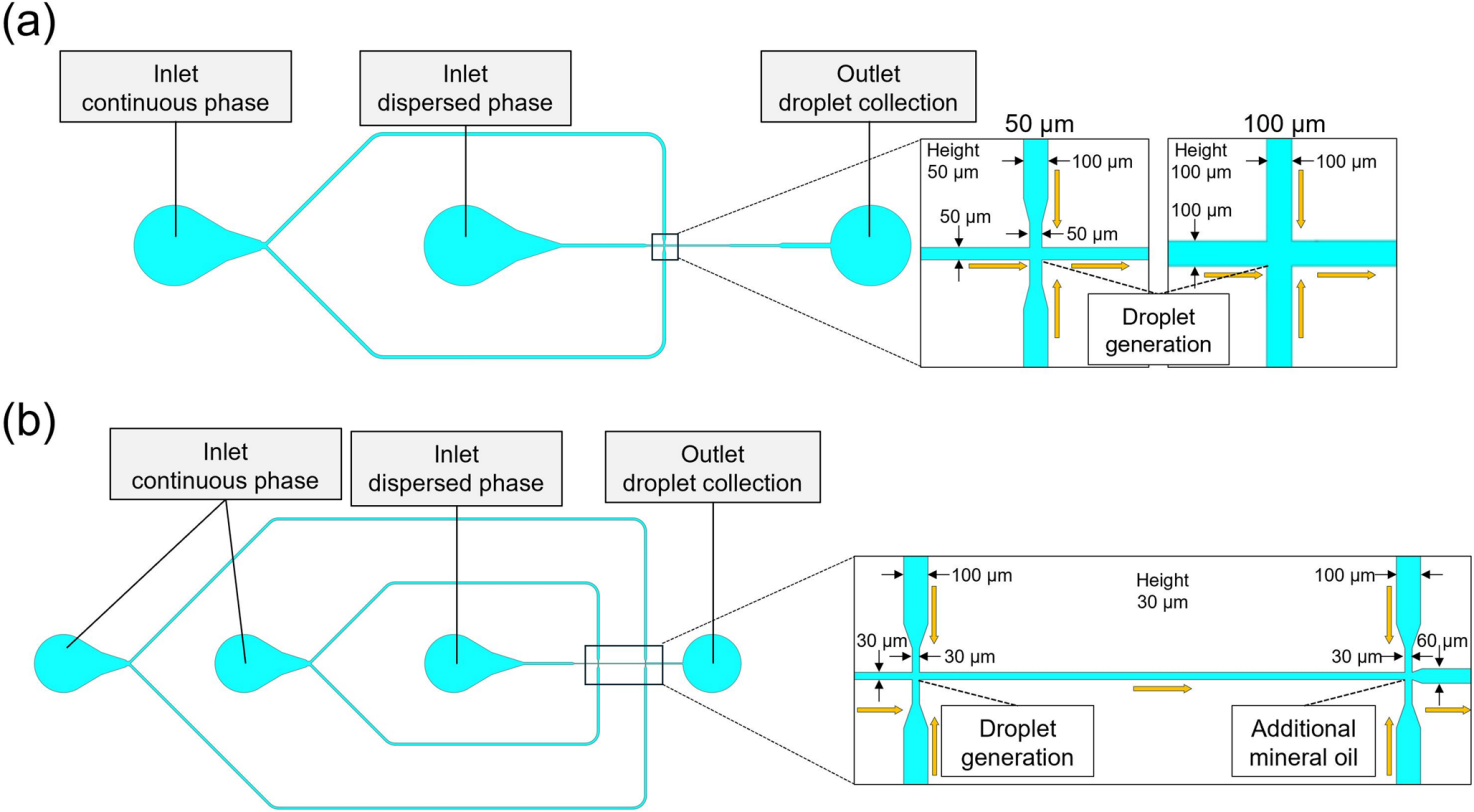
Device designs and dimensions for droplet generation. (**a**) Schematic of the channel for generating droplets with a diameter of 50 or 100 μm. (**b**) Schematic of the channel for generating droplets with a diameter of 30 μm. Mineral oil was injected through the left inlet to maintain the distance between droplets. Orange arrows indicate the direction of solution

The channel design is shown in Fig. 1. The device had two or three inlets to inject the continuous- or dispersed-phase solutions, respectively. Additionally, the device featured a cross-junction area for generating droplets using a flow-focusing system. The flow-focusing junction width and the uniform channel height were both set to the nominal target droplet diameter for each device. For 30 μm droplets, a side channel was incorporated to prevent droplet fusion by maintaining the distance between each droplet. The devices were fabricated from PDMS using the soft lithography method. To create the mold, the channel design was patterned onto an SU-8 resist (SU-8 3025 or 3050, Kayaku Advanced Materials, Inc., Westborough, MA, USA) on a silicon wafer using a maskless aligner (MLA150, Heidelberg Instruments, Heidelberg, Germany). After development, eyelets for the inlet and the outlet were glued to the substrate. Then, after plasma bonding of PDMS layer (into which the channel pattern had been transferred) to a glass slide, Cytop-809M (AGC Chemicals, Tokyo, Japan) was injected into the devices and they were heated at 200 °C for 8 h to make the channel surface hydrophobic.

### Droplet generation system setup

**Fig. 2:**
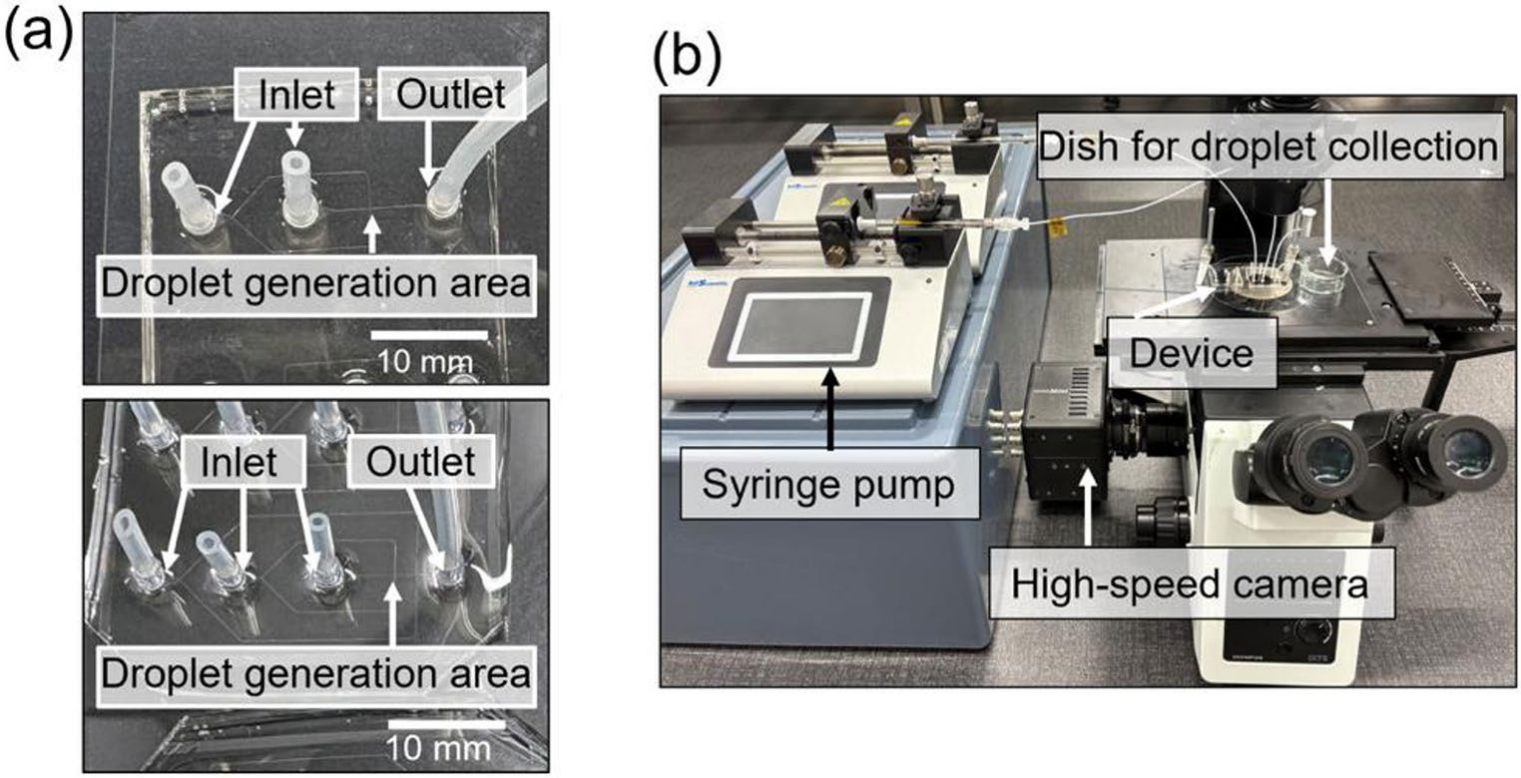
Microfluidic device as used in the experiments. (**a**) Photograph of the PDMS/glass device for generating 50 and 100 μm droplets (upper) and 30 μm droplets (lower), respectively. (**b**) Droplet generation experimental system setup

Figure 2a shows a photograph of the PDMS microfluidic device. The droplet generation experimental setup is shown in Fig. 2b. The continuous- and dispersed-phase solutions were injected thorough the inlets of the device using syringes (1725CX or 1750CX, Hamilton, Reno, NV, USA) and syringe pumps (LEGATO 101 , KD Scientific Inc., Holliston, MA, USA). Flow in the channel was observed using an optical microscope (IX71, Olympus, Tokyo, Japan) and a high-speed camera (FASTCAM Mini UX50, Photron, Tokyo, Japan). The droplets were collected from the outlet in a glass dish containing mineral oil.

### Agarose droplet generation and counting

A 1 wt % agarose solution was prepared for droplet generation. Agarose was dissolved in Milli-Q water for encapsulating fluorescence particles, and in phosphate-buffered saline (PBS) for *E. coli* cells. Mineral oil containing 1 wt % Span 80 was used as the carrier oil. Agarose solution was mixed with 1 μm fluorescent beads or *E. coli* cells, and the particle concentration was adjusted based on Poisson loading statistics to target an expected occupancy of λ = 1 for each droplet diameter. Assuming spherical droplets with diameter *d*, the droplet volume *V* was calculated as *V* = (π/6) *d*³. The required particle concentration *C* was then calculated as *C* = λ/*V*. The flow rate of the syringe pumps for each droplet size was set as shown in Table 1. Droplet size was adjusted by tuning the flow rate of the continuous phase and the width and height of the channel. The droplets exited from the device through the outlet and were collected in a glass dish containing mineral oil to prevent droplet evaporation. The droplets were left to sit at 4 °C for 30 min to form a stable gel and then were observed using confocal laser scanning microscopy (FV3000, Evident, Tokyo, Japan) to count the droplets encapsulating the particles.

**Table 1:** Flow rates of the continuous phase and dispersed phase for each droplet size

Droplet diameter (µm)	Mineral oil flow rate (µL/min)	Agarose flow rate (µL/min)
30	2.5 or 3.0	0.5
50	3.3 or 3.8	0.5
100	7.0	0.5

### Statistical evaluation of droplet encapsulation

For droplets encapsulating particles, the agreement between the experimentally observed encapsulation distribution and the corresponding Poisson prediction was quantitatively evaluated using the coefficient of determination (*R²*) and the mean absolute error (MAE), which capture the overall goodness of fit and the average absolute deviation, respectively. Let $\:{n}_{k}\:$ be the experimentally observed number of droplets containing $\:k\:\mathrm{p}\mathrm{a}\mathrm{r}\mathrm{t}\mathrm{i}\mathrm{c}\mathrm{l}\mathrm{e}\mathrm{s}\:(k=0,\:1,\:2,\dots\:,\:{K})$, and $N_{total}$ be the total number of droplets analyzed. Let $\:{\widehat{n}}_{k}$ be the Poisson-predicted expected number of droplets containing $k$ particles, calculated from Eq. (1) and multiplied by $N_{total}$. Here, *K* denotes the maximum number of occupancy category included in the analysis; in this study, droplets containing up to six particles (*K* = 6) were observed and included in the statistical evaluation. 

*R²* was calculated as

$$\:{R}^{2}=1-\frac{{\sum\:}_{k=0}^{K}{\left({n}_{k}-{\widehat{n}}_{k}\right)}^{2}}{{\sum\:}_{k=0}^{K}{\left({n}_{k}-\stackrel{-}{n}\right)}^{2}} \, ,$$

where $$\:\stackrel{-}{n}=\frac{1}{K+1}{\sum\:}_{k=0}^{K}{n}_{k}$$.

MAE was defined as

$$\:\mathrm{M}\mathrm{A}\mathrm{E}=\frac{1}{K+1}{\sum\:}_{k=0}^{K}\left|{n}_{k}-{\widehat{n}}_{k}\right| \, .$$

### *E. coli* preparation

*E. coli* cells were used as a biological sample. The *E. coli* strain JM109 was transformed with the plasmid pUC18-GFP (Nippon Gene, Tokyo, Japan). Transformed cells were plated on Luria–Bertani (LB) agar containing ampicillin and isopropyl β-D-thiogalactopyranoside (IPTG), and incubated overnight at 37 °C. A single colony was picked and inoculated into LB broth (5 mL) supplemented with ampicillin, followed by overnight incubation at 37 °C. Subsequently, IPTG was added to a final concentration of 0.1 mM, and the culture was incubated for an additional 3 h at 37 °C with shaking at 150 rpm to induce GFP expression. *E. coli* was harvested and resuspended in PBS buffer. The cell concentration was determined using a bacterial cell counter. The mixture of agarose and cell culture medium was adjusted for droplet generation to achieve the target droplet diameters.

## Results and discussion

### Droplet generation and collection

**Fig. 3:**
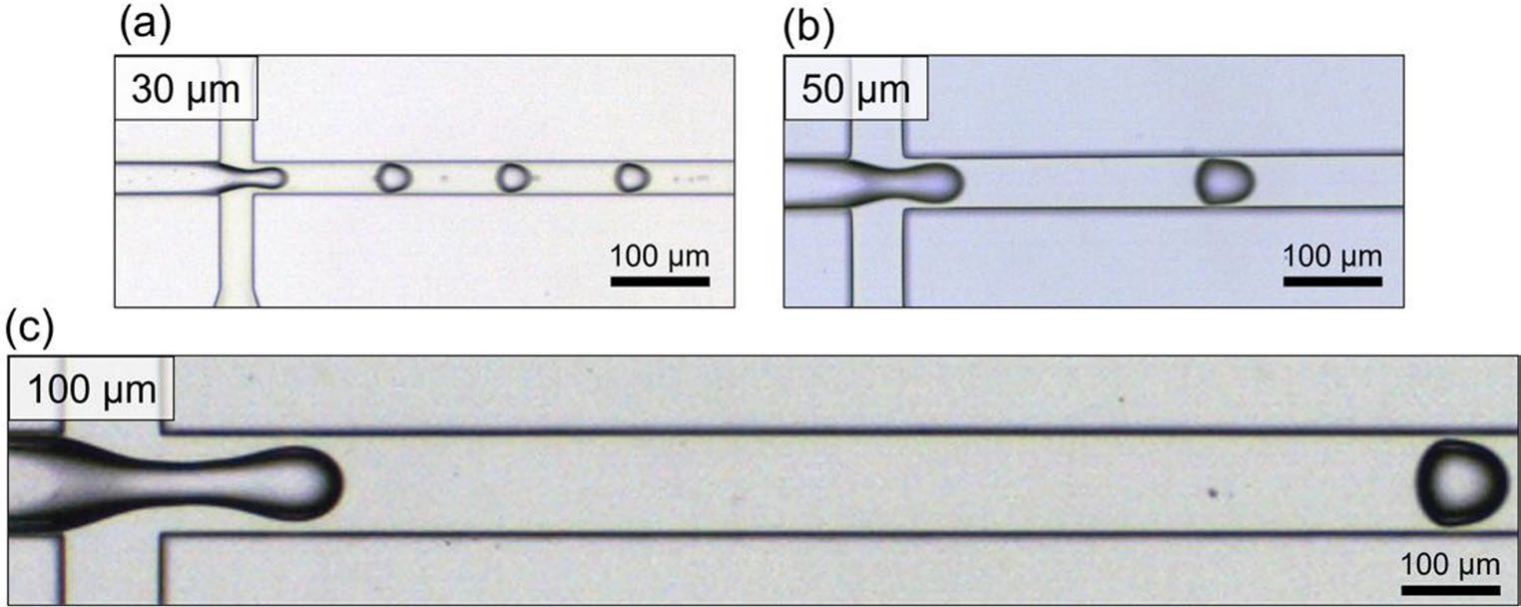
Micrograph of droplet generation in the flow-focusing area of the microfluidic device for (**a**) 30, (**b**) 50, and (**c**) 100 μm diameter droplets

Figure 3 shows images of the flow-focusing area of the device and shows the flow of agarose. The height and width of the devices in the flow-focusing were varied to generate each droplet size (30, 50, and 100 μm). The width of the flow channel near the outlet was designed to be wider than that of the cross-junction area to prevent the droplets merging at the outlet, where the flow width increased substantially. However, the device for 30 μm droplets was modified by adding a channel that merged mineral oil into the main flow after the cross-junction area to maintain the distance between droplets and prevent them from merging (Fig. 1b). Agarose, which was used as the dispersed phase, was sheared by the mineral oil continuous phase. For each droplet diameter, the particle concentration was adjusted to target λ = 1 based on the calculated values summarized in Supplementary Table S1.The flow rates required for droplets generation depended on the target droplet diameter (Table 1) and were determined through preliminary calibration experiments. In these experiments, the dispersed-phase flow rate (0.5 µL/min), fluid composition, and temperature were kept constant, while the continuous-phase flow rate was varied to identify operating conditions that reproducibly generated the desired droplet sizes. Based on real-time microscopic observation of droplet formation, flow conditions yielding stable generation at the target diameters were selected for the encapsulation experiments. All operating conditions remained within the dripping regime, as evidenced by the stable and periodic pinch-off observed in the flow-focusing region (Supplementary Videos 1–3). Droplets of the desired size were consistently generated with these devices.

### Encapsulation efficiency at each droplet diameter

**Fig. 4:**
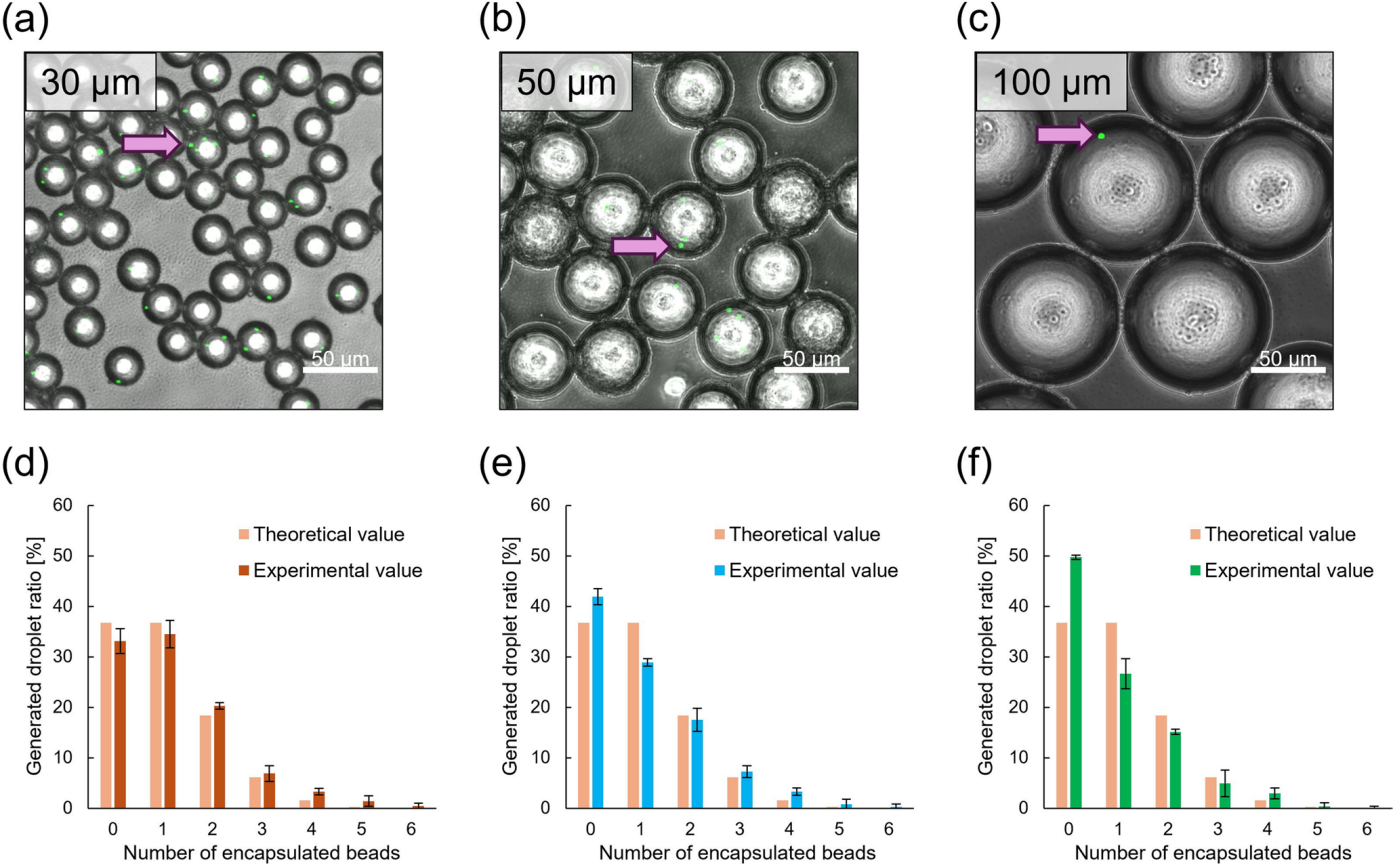
Collected droplets of each size and bar graphs showing the number of encapsulated beads in droplets of different sizes. Micrographs of droplets measuring (a) 30, (b) 50, and (c) 100 µm. Pink arrows indicate the fluorescence beads. Bar graphs showing distributions of the number of encapsulated beads for (d) 30, (e) 50, and (f) 100 µm diameter droplets. Error bars represent the standard deviation of three independent experiments (n = 3)

Figure 4a–c shows micrographs of droplets 30, 50, and 100 μm in diameter after cooling. Adjustment of the device design and the flow rate of the continuous phase allowed droplets of each size to be generated as expected. The green particles in the micrographs are 1 μm fluorescent beads encapsulated within droplets. Approximately 200–250 droplets were randomly selected from the transmitted light detector (TD) micrographs prior to overlaying the fluorescence images, and only droplets within ± 5% of the target diameter were included in the analysis.

Figure 4d–f shows the distributions of the number of encapsulated particles in droplets with different diameters. Supplementary Table S2 summarizes the quantitative assessment metrics (*R²* and MAE) and the total number of droplets analyzed in each independent experiment. For the 30 μm droplets, the observed frequencies closely followed the theoretical Poisson distribution (λ = 1), with *R*^2^ consistently exceeding 0.95 (Fig. 4d). The 50 μm droplets also showed broad agreement with the Poisson model, with *R²* values above 0.92 (Fig. 4e). A quantitative assessment of how closely the droplet encapsulation followed the Poisson distribution was performed using the MAE, which measures the average magnitude of absolute deviations between observed and expected frequencies. The 30 μm droplets exhibited smaller overall discrepancies with an average MAE of 3.81, whereas 50 μm droplets showed a higher average MAE of 5.62, primarily due to an excess of empty droplets and a lower-than-expected frequency of single-particle droplets. These results indicate that although both droplet sizes generally follow Poisson statistics, smaller droplets tended to provide a closer fit with reduced distributional deviations.

In contrast, the encapsulation behavior of 100 μm droplets differed notably from that of smaller droplets (Fig. 4f). In all experimental replicates, the *R*^2^ values remained below 0.89, and the mean MAE reached 10.9, indicating substantial deviations from the theoretical distribution. Approximately half of the droplets were empty, whereas only around 26% contained a single particle. These observations show that the extent of deviation from Poisson statistics increased with droplet diameter, suggesting that larger droplets were associated with reduced encapsulation fidelity under passive conditions.

To examine whether deviations from the nominal λ = 1 could influence the observed trends, the experimental λ (λ_exp) was calculated for each dataset based on the total number of encapsulated particles divided by the total number of droplets. The corresponding theoretical Poisson distributions were generated and compared with the experimental data. The results remained consistent with the original analysis: droplets of 30 μm and 50 μm exhibited encapsulation distributions that aligned well with their respective Poisson predictions (Fig. S1a, b), whereas 100 μm droplets continued to show pronounced deviations (Fig. S1c). Thus, the discrepancies observed for 100 μm droplets cannot be attributed to inaccuracies in λ estimation, but instead reflect effects arising from droplet volume and the associated flow conditions.

Several physical and fluidic factors may contribute to the deviations from the Poisson distribution, particularly for the 100 μm droplets. Under passive flow without active mixing, gravitational settling and local stagnation can occur, especially in larger microchannels, such as those 100 μm high and wide. Previous investigations into cell encapsulation in droplets have demonstrated that sedimentation and aggregation skew distribution significantly, with higher fluid viscosity or density matching improving uniformity (Puttrich et al. 2023; Pranauskaite et al. 2024). Furthermore, the larger volume of 100 μm droplets reduces the effective particle concentration per droplet, making them more sensitive to any pre-existing heterogeneity in the sample. In contrast, smaller droplets (30–50 μm) inherently confine particles within a smaller volume and are formed in narrower microchannels, conditions that help maintain uniform particle dispersion before droplet pinch-off, leading to more predictable, Poisson-like encapsulation.

We note that different channel geometries were used to generate different droplet sizes, which may influence local flow fields and breakup dynamics. However, all experiments were performed under stable dripping conditions using identical fluid compositions and target occupancies (λ = 1). The present study therefore focuses on identifying practical size-dependent trends in encapsulation behavior rather than fully isolating geometric effects.

These results highlight the relevance of droplet size control for workflows that rely on controlled particle or cell loading. In applications such as single-cell assays, digital PCR, and functional screening, achieving predictable occupancy contributes to reliable quantification and reduced variability. Our bead-based experiments suggest that decreasing droplet diameter can improve the agreement with Poisson-based encapsulation under passive condition, without the need for external focusing or active sorting mechanisms. This simplicity may be advantageous for research seeking straightforward device operation and minimal instrumentation. Taken together, these observations indicate that droplet diameter is an important parameter influencing encapsulation behavior in passive systems, while the extent of its impact may depend on the particle type and experimental context. These findings provide practical guidance for the design and optimization of droplet-based workflows, and warrant further evaluation using biological samples.

### Application to *E. coli* cells with encapsulating experiments

**Fig. 5:**
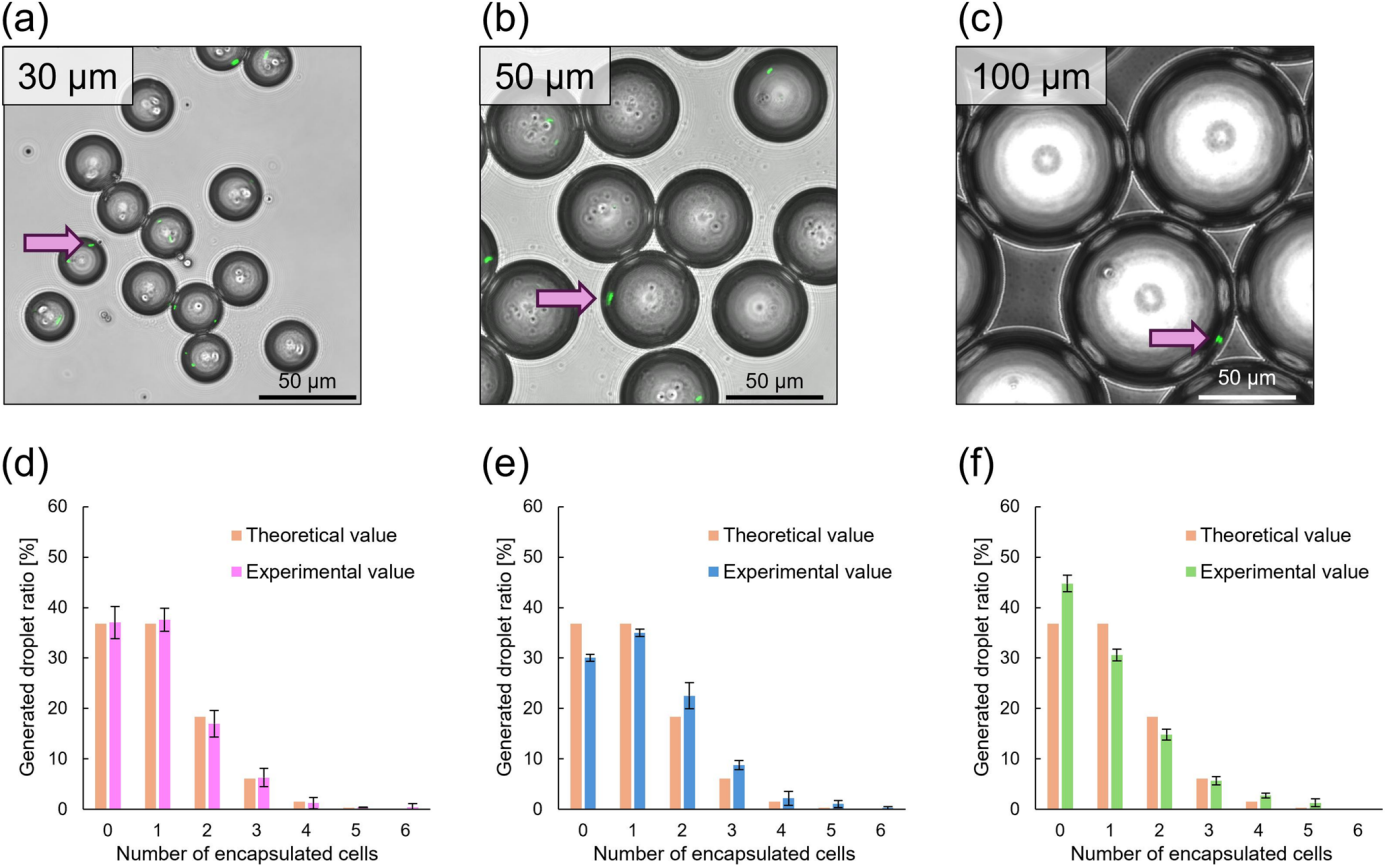
Representative micrographs of droplets encapsulating GFP-expressing *E. coli* at different droplet diameters and the corresponding occupancy distributions. (**a**-**c**) Droplets with diameters of 30, 50, and 100 μm are shown, respectively. *E. coli* cells are indicated by pink arrows. (**d**-**f**) The distributions of the number of encapsulated *E. coli* per droplet for 30, 50, and 100 μm droplets, respectively. Error bars denote the standard deviation obtained from three independent experiments (*n* = 3)

To evaluate whether the encapsulation behavior of cells shows comparable size-dependent trends to those observed with fluorescent beads, GFP-expressing *Escherichia coli* cells were encapsulated in agarose droplets of 30, 50, and 100 μm in diameter under the same target occupancy (λ = 1). *E. coli* cells typically exhibit a rod-like morphology with a length of approximately 2 μm and a diameter of ~ 0.8 μm, depending on strain and growth conditions (Männik et al. 2009; Osiro et al. 2012). These dimensions are comparable to those of the 1 μm fluorescent beads used in this study in terms of characteristic length scale, while exhibiting greater morphological heterogeneity. The dispersed phase consisted of agarose mixed with PBS containing GFP-expressing *E. coli*. Because PBS is more viscous than distilled water, the flow rate of the continuous phase was adjusted to ensure sufficient shear force to generate droplets of the target diameters. Droplets encapsulating *E. coli* were then generated and observed (Fig. 5a-c).

Figure 5d–f presents the occupancy distributions of *E. coli* encapsulated in droplets of different diameters. Supplementary Table S3 provides the number of analyzed droplets and the corresponding quantitative assessment metrics (*R²* and MAE) for each experiment. For the 30 μm droplets, the experimental distributions showed close agreement with the theoretical Poisson model (λ = 1), with *R²* values consistently above 0.97 (Fig. 5d). In comparison, the 50 μm and 100 μm droplets showed generally good agreement with the Poisson prediction, with *R²* values in the ranges of approximately 0.92–0.95 and 0.91–0.96, respectively (Fig. 5e, f). These results indicate that while the closest agreement was achieved for the smallest droplets, the overall goodness of fit remained comparable between the 50 and 100 μm droplets. The MAE values for all droplet sizes were within a similar range (approximately 2.3–7.0), indicating that the overall magnitude of absolute deviations from the Poisson prediction was comparable across the investigated droplet diameters. The experimentally estimated occupancy (λ_exp) was additionally calculated to account for possible deviations from the nominal λ = 1 when interpreting the encapsulation behavior. For the 30 μm and 50 μm droplets, the experimentally observed occupancy distributions were generally consistent with the corresponding Poisson predictions based on λ_exp　(Fig. S2a, b). In contrast, for the 100 μm droplets, a higher fraction of empty droplets and a lower-than-expected frequency of single-cell occupancy were observed relative to the Poisson prediction (Fig. S2c). Although these deviations were moderate compared with the bead-based experiments, they suggest that larger droplets tend to exhibit a higher fraction of empty droplets and slight deviations from ideal stochastic loading.

Interestingly, while *E. coli* encapsulation exhibited a broadly similar trend to the bead experiments in that smaller droplets showed the closest agreement with Poisson statistics, the size-dependent deterioration was less pronounced for *E. coli*, and the 50 and 100 μm droplets remained comparable in goodness of fit. This difference may arise from several non-exclusive factors. Hydrodynamic ordering or particle correlations upstream of droplet pinch-off can modify particle-arrival statistics and lead to deviations from ideal Poisson loading, and non-uniform particle delivery such as sedimentation or aggregation may further alter effective loading concentrations (Abate et al. 2009; Bithi and Vanapalli 2017). Because rigid monodisperse beads and rod-shaped biological cells vary in size distribution, shape, surface properties, and deformability, the degree of such flow-induced correlations may differ between particle types. In addition, image-based identification and counting of bacteria introduce intrinsic uncertainty arising from variability in fluorescence and morphology, which may partially mask subtle size-dependent differences in quantitative agreement metrics (Sesen and Whyte 2020). Taken together, these considerations suggest that the reduced size-dependent divergence observed for *E. coli* reflects a combination of transport effects, flow-induced correlations, and measurement uncertainty, rather than droplet size alone. In the present study, particles in the 1–2 μm size range were selected to represent microorganism-scale samples and to maintain a sufficiently small particle-to-droplet size ratio such that Poisson-based stochastic loading remains a reasonable baseline for quantitative comparison across droplet diameters. For substantially larger particles or cells, additional physical effects such as excluded volume, geometric confinement, and size-dependent transport may become increasingly significant and alter encapsulation statistics.

These findings indicate that passive droplet generation at small diameters (e.g., 30 μm) can reproducibly yield stochastic encapsulation patterns that are well described by Poisson statistics for both rigid beads and bacterial cells. Under stable dripping conditions, small droplets therefore provide a practical approach for achieving reliable stochastic loading for single particle/cell encapsulation.

## Conclusion

The effect of droplet size on stochastic encapsulation behavior was systematically investigated using agarose droplets with diameters of 30, 50, and 100 μm in a passive flow-focusing microfluidic system. Encapsulation performance was quantitatively evaluated using statistical agreement metrics for fluorescent beads and GFP-expressing *Escherichia coli* cells under a target occupancy of λ = 1.

For fluorescent beads, smaller droplets exhibited closer agreement with Poisson statistics, whereas 100 μm droplets showed pronounced deviations characterized by an increased fraction of empty droplets. In contrast, *E. coli* encapsulation followed a broadly similar size-dependent trend, with the closest agreement observed for 30 μm droplets, while the 50 and 100 μm droplets maintained comparable encapsulation fidelity and moderate deviations, including a modest increase in empty droplets at the largest diameter. These results indicate that droplet size is an important parameter governing stochastic encapsulation performance in passive systems, although the magnitude of its effect depends on particle type, and that smaller droplets generally provide more robust and reproducible encapsulation behavior across both inert particles and biological cells. At the same time, the particle-dependent differences observed here suggest that encapsulation behavior could not be inferred solely from bead-based model systems. The present study is limited to passive droplet generation using a limited set of channel geometries and representative particle types, and further studies may help clarify how geometric and particle-dependent factors interact with droplet-size-dependent encapsulation behavior.

Overall, our findings suggest that careful optimization of droplet size is an important factor for achieving reliable stochastic encapsulation in simplified passive microfluidic platforms, and can help inform the design of droplet-based assays for applications such as digital PCR and single-cell analysis.

## Supplementary Information

Below is the link to the electronic supplementary material.

Supplementary Video 1

Supplementary Video 2

Supplementary Video 3

Supplementary Material

## Data Availability

The data supporting the findings of this study are available from the corresponding author upon reasonable request.
