# Supplementary material for: Effect of droplet size on the encapsulation efficiency of microparticles in passive microfluidic systems
Source: Biomed Microdevices. 2026 Mar 31;28(2):26. doi: 10.1007/s10544-026-00795-0 (PMC13038637; doi:10.1007/s10544-026-00795-0)
Supplement: Supplementary file 4 — Supplementary Material [file 10544_2026_795_MOESM4_ESM.docx]

**Supplementary material**

**Effect of Droplet Size on the Encapsulation Efficiency of Microparticles in Passive Microfluidic Systems**

Risa Fujita^1^, Masashi Kobayashi^2^, Shuichi Shoji^2^, Takashi Tanii^1,2^, Masahiro Furuya^2^ and Daiki Tanaka^2*^

1. Research Organization for Nano & Life Innovation, Waseda University, Tokyo, JAPAN

2. Faculty of Science and Engineering, Waseda University, Tokyo, JAPAN

***Corresponding author:**

Daiki Tanaka

Address: Faculty of Science and Engineering, Waseda University, Tokyo 169-8555, Japan

Phone: +81-3-5286-9067

Email: [d.tanaka@ruri.waseda.jp](mailto:d.tanaka@ruri.waseda.jp)

**
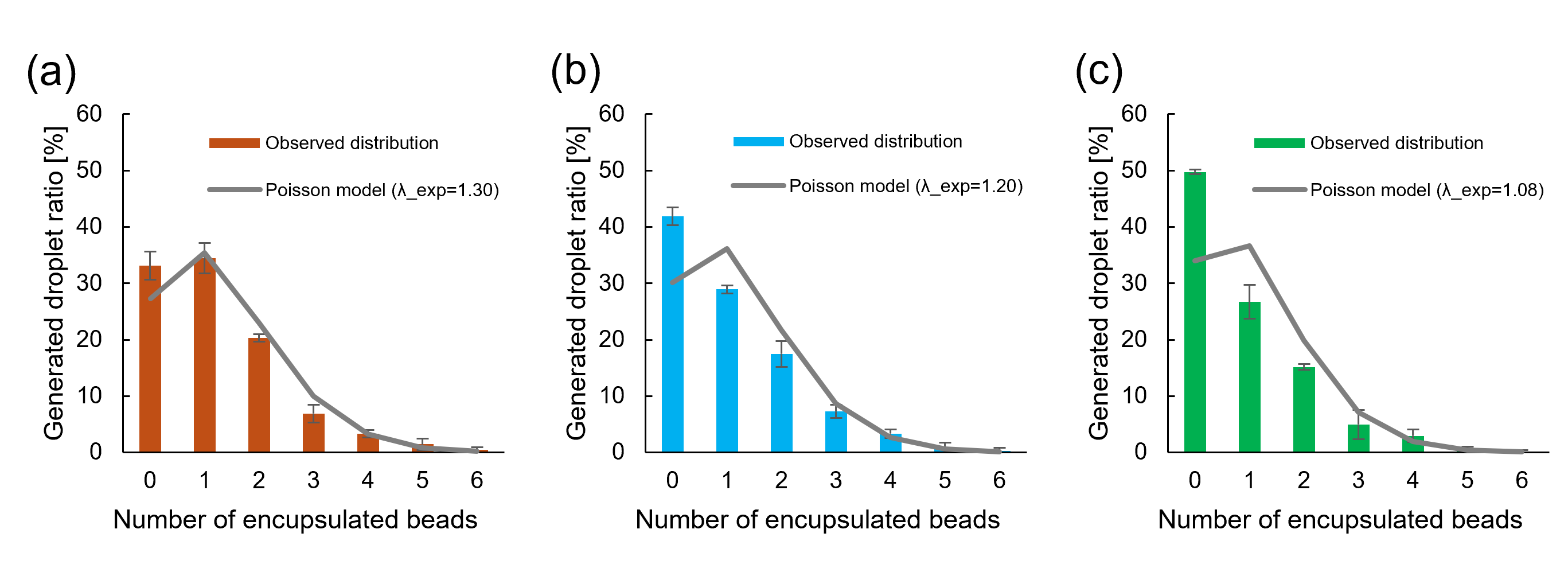
Figure S1.** Comparison of experimental encapsulation distributions with Poisson model predictions for droplets encapsulating fluorescent beads of (a) 30 µm, (b) 50 µm, and (c) 100 µm in diameter. Bars show the mean ± standard deviation from three independent experiments. Solid lines represent the theoretical Poisson distributions calculated using the averaged λ_exp values for each droplet size.


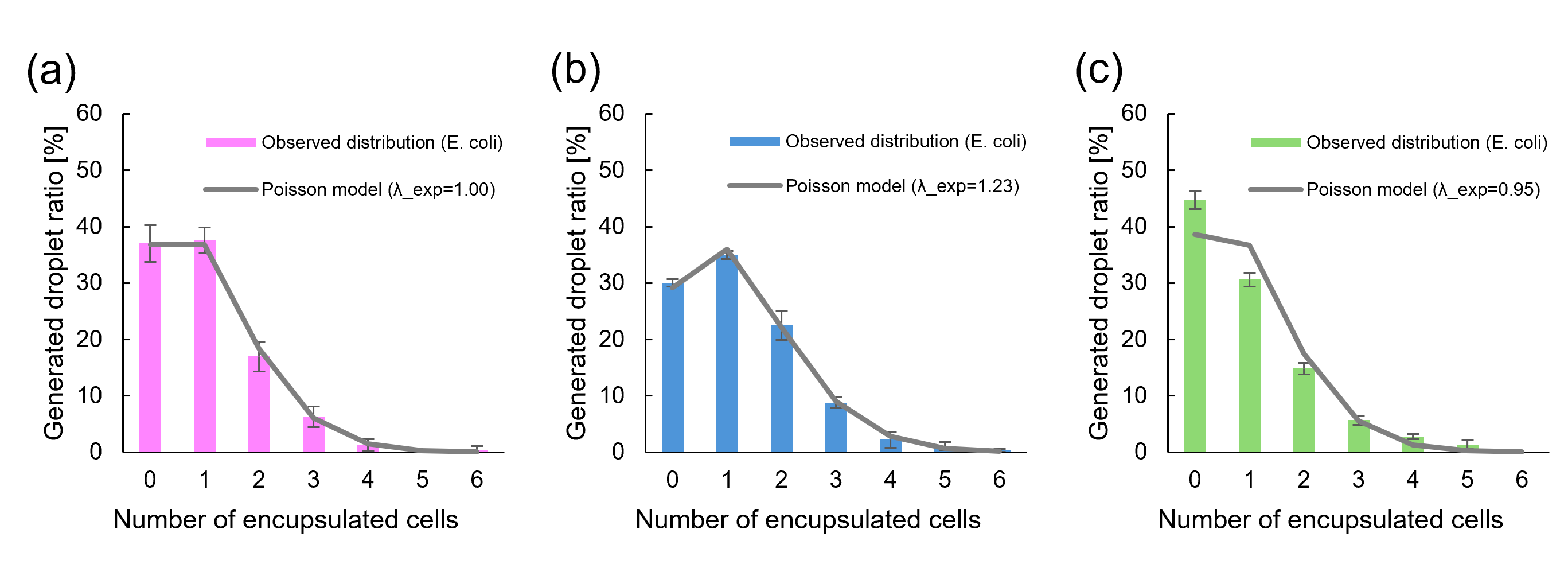


**Figure S2.** Comparison of experimental encapsulation distributions with Poisson model predictions for droplets encapsulating *E. coli* cells of (a) 30 µm, (b) 50 µm, and (c) 100 µm in diameter. Bars show the mean ± standard deviation from three independent experiments. Solid lines represent the theoretical Poisson distributions calculated using the averaged λ_exp values for each droplet size.

**Table S1.** Calculated particle concentrations for λ = 1 at each droplet diameter

| **Droplet size (µm)** | **Nominal droplet volume (pL)** | **Target λ** | **Calculated concentration (particles/mL)** |
| --- | --- | --- | --- |
| 30 | 14.1 | 1.0 | 7.09 × 10⁷ |
| 50 | 65.4 | 1.0 | 1.53 × 10⁷ |
| 100 | 523.6 | 1.0 | 1.91 × 10⁶ |

**Table S2.** Quantitative assessment of encapsulation fidelity for fluorescent beads encapsulation experiments

| **Droplet size**  **(µm)** | **Experiment No.** | **The number of droplets** | **MAE** | ***R²*** | **λ_exp** |
| --- | --- | --- | --- | --- | --- |
| 30 | 1 | 204 | 4.60 | 0.960 | 1.26 |
| 30 | 2 | 233 | 2.53 | 0.991 | 1.27 |
| 30 | 3 | 208 | 4.30 | 0.956 | 1.37 |
| 50 | 1 | 213 | 6.76 | 0.924 | 1.12 |
| 50 | 2 | 217 | 5.83 | 0.928 | 1.25 |
| 50 | 3 | 200 | 4.27 | 0.95 | 1.23 |
| 100 | 1 | 237 | 9.44 | 0.883 | 1.05 |
| 100 | 2 | 253 | 12.50 | 0.804 | 1.15 |
| 100 | 3 | 250 | 10.63 | 0.870 | 1.03 |

**Table S3.** Quantitative assessment of encapsulation fidelity for *E. coli* encapsulation experiments

| **Droplet size**  **(µm)** | **Experiment No.** | **The number of droplets** | **MAE** | ***R²*** | **λ_exp** |
| --- | --- | --- | --- | --- | --- |
| 30 | 1 | 231 | 4.38 | 0.979 | 0.87 |
| 30 | 2 | 240 | 2.32 | 0.994 | 1.01 |
| 30 | 3 | 240 | 2.78 | 0.992 | 1.13 |
| 50 | 1 | 208 | 5.81 | 0.924 | 1.18 |
| 50 | 2 | 215 | 5.48 | 0.936 | 1.29 |
| 50 | 3 | 203 | 4.39 | 0.952 | 1.21 |
| 100 | 1 | 211 | 6.45 | 0.928 | 0.94 |
| 100 | 2 | 201 | 6.96 | 0.911 | 0.94 |
| 100 | 3 | 202 | 4.85 | 0.957 | 0.97 |
